# Negative Marital Quality, Purpose in Life, and Depressive Symptoms Among Middle-Aged and Older Couples

**DOI:** 10.1093/geroni/igaa057.1609

**Published:** 2020-12-16

**Authors:** Elliane Irani, Sumin Park, Ronald Hickman

**Affiliations:** 1 Case Western Reserve University, Ohio, United States; 2 Case Western Reserve University, Cleveland Heights, Ohio, United States; 3 Case Western Reserve University, Cleveland, Ohio, United States

## Abstract

Negative marital quality is associated with poor health outcomes. Purpose in life can serve as a psychological resource to buffer the stressors experienced from a negative marital relationship. Yet, the associations among negative martial quality, a person’s level of purpose in life, and depressive symptoms have not been fully explored in a dyadic context. We examined the actor (intra-individual) and partner (cross-spousal) effects of negative marital quality on depressive symptoms in couples and the potential mediating role of purpose in life. Structural equation modeling was used to analyze cross-sectional data on middle-aged and older married, heterosexual couples (N=1,235) who participated in the 2016 wave of the Health and Retirement Study. The final model had an acceptable fit to the data (TLI=.963, RMSEA=.040, SRMR=.038). At the actor level, negative relationship quality was positively associated with depressive symptom severity, and purpose in life mediated the relationship in wives and husbands. At the partner level, wives had more depressive symptoms when husbands reported higher negative marital quality. Comparatively, husbands had less depressive symptoms when their wives indicated a greater sense of purpose. Husbands also had a lower purpose in life when their wives had higher states of negative marital quality. This study highlights the psychological benefits of allaying negative perceptions of marital quality and enhancing the sense of purpose in middle-aged and older couples. The results support a focus on dyadic approaches to improve the psychological health, and potentially, the physical health status of middle-aged and older couples.

